# Advancing Heart Failure Care Through Disease Management Programs: A Comprehensive Framework to Improve Outcomes

**DOI:** 10.3390/jcdd12080302

**Published:** 2025-08-05

**Authors:** Maha Inam, Robert M. Sangrigoli, Linda Ruppert, Pooja Saiganesh, Eman A. Hamad

**Affiliations:** Department of Medicine, Lewis Katz School of Medicine, Temple University, Philadelphia, PA 19147, USA

**Keywords:** heart failure, disease management program, guideline-directed medical therapy, patient education, multidisciplinary care, health equity, remote monitoring, heart failure readmission

## Abstract

Heart failure (HF) is a major global health challenge, characterized by high morbidity, mortality, and frequent hospital readmissions. Despite the advent of guideline-directed medical therapies (GDMTs), the burden of HF continues to grow, necessitating a shift toward comprehensive, multidisciplinary care models. Heart Failure Disease Management Programs (HF-DMPs) have emerged as structured frameworks that integrate evidence-based medical therapy, patient education, telemonitoring, and support for social determinants of health to optimize outcomes and reduce healthcare costs. This review outlines the key components of HF-DMPs, including patient identification and risk stratification, pharmacologic optimization, team-based care, transitional follow-up, remote monitoring, performance metrics, and social support systems. Incorporating tools such as artificial intelligence, pharmacist-led titration, and community health worker support, HF-DMPs represent a scalable approach to improving care delivery. The success of these programs depends on tailored interventions, interdisciplinary collaboration, and health equity-driven strategies.

## 1. Introduction

Heart failure (HF) remains a leading cause of morbidity and mortality globally. Around 6.7 million people over 20 years of age have HF in the U.S., and this number is expected to rise to 8.7 million by 2030 [1]. HF mortality rates have also been on the rise and accounted for 45% of all cardiovascular related deaths in the U.S. in 2021 [1]. In addition to its clinical impact, HF imposes a significant economic burden, costing the U.S. healthcare system an estimated USD 30.7 billion annually, with hospitalizations responsible for nearly 80% of these costs. Recurrent hospitalizations not only contribute to lost productivity and reduced quality of life but also result in financial penalties for hospitals under the Centers for Medicare & Medicaid Services (CMS) Hospital Readmissions Reduction Program.

Despite the development of advanced goal-directed medical therapies (GDMTs), there is a constant rise in HF morbidity and mortality. The management and treatment of HF requires complex multidisciplinary care with many teams in collaboration, both inpatient and outpatient, and proves to be a growing health and economic burden [2].

Several new models have been developed for managing and coordinating the increasing complexity of care for HF patients [3]. These are aimed at replacing traditional approaches that often solely focus on the medical aspect of patient care and instead incorporate a multifaceted approach accounting for the social determinants of health (SDoH) in the treatment approach. The aim behind this strategy is twofold; to improve healthcare outcomes and reduce costs for healthcare systems. An integral aspect of disease management interventions is HF education for patients and their families, along with coordinated follow-up clinic visits and testing. These initiatives have been shown to enhance patients’ understanding of their illness and subsequently improve patient outcomes.

**Figure 1:**
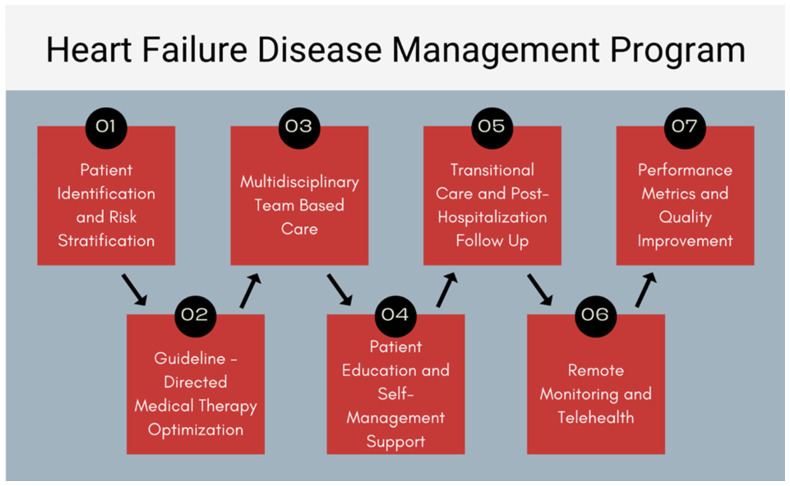
Essential components of a heart failure disease management program.

The success and efficacy of Heart Failure disease management programs (HF-DMPs) in achieving these goals remain uncertain and greatly vary across health systems and target populations. This review paper aims to outline the development process of a HF-DMP, as well as its essential components and intended outcomes (Figure 1).

## 2. Patient Identification and Risk Stratification

Patient risk stratification is critical to initiate early interventions that may prevent adverse outcomes and reduce healthcare utilization [4]. There have been numerous methods and strategies adopted to predict hospital admission in patients with HF, and these have rapidly evolved with changes in clinical guidelines and advancements in technology [5]. Electronic health records (EHRs) are one of the primary valuable tools in risk prediction, enabling the identification of high-risk HF patients through the analysis of routinely collected clinical data [6]. Biomarkers have also played a central role in HF risk stratification; elevated N-terminal pro-B-type natriuretic peptide (NT-proBNP) levels, particularly above 125 pg/mL, have been associated with a 2.4-fold increased risk of incident HF, and its predictive value is enhanced when combined with high-sensitivity cardiac troponin [7,8]. Nevertheless, biomarker use is limited by the need for frequent lab draws that may not be readily accessible in all care settings [9,10]. Serial echocardiography, specifically monitoring left ventricular function and left atrium structure, has also been shown to improve risk stratification, underscoring the need for repeated assessment in patients with HF [11,12].

Several validated risk prediction models have been developed to aid clinicians in identifying patients at the highest risk of adverse outcomes. The Meta-Analysis Global Group in Chronic Heart Failure (MAGGIC) score predicts long-term all-cause mortality in chronic HF patients, while the LACE index estimates both the 30-day readmission and mortality risk in patients with HF [13,14,15]. While the MAGGIC score has been an effective tool for predicting all-cause mortality in ambulatory populations, its performance and predictive accuracy decline when applied at hospital discharge [16]. In a comparative analysis, the LACE index demonstrated a moderate ability to predict 30-day readmissions with an area under the receiver operating curve (AUC) of 0.73, similar to the HOSPITAL score (AUC 0.69; *p* ≤ 0.001) [17]. Although both these tools assist in broad risk stratification, they are limited by static data inputs and may fail to capture dynamic, real-time clinical changes. 

Specifically, we now emphasize that risk stratification tools—such as the LACE index, the Seattle Heart Failure Model, and other institution-specific algorithms—play a pivotal role in triaging patients based on their likelihood of readmission or clinical deterioration. These scores are often embedded within the electronic health record and reviewed by care teams at key transition points (e.g., hospital discharge, emergency department visit, or new diagnosis of HF).

These tools inform the intensity and frequency of follow-up within HF-DMPs. For example, patients identified as high-risk may be flagged for early post-discharge clinic visits (within 72 h), increased telephone surveillance by heart failure nurse coordinators, or prioritization for home telemonitoring. In contrast, lower-risk patients may be managed with standard clinic follow-up and education reinforcement. In the acute care setting, these models can help determine whether a patient requires inpatient admission, overnight observation, or can be safely managed as an outpatient with close follow-up. In outpatient HF clinics, elevated risk scores may prompt earlier in-person assessment or lab testing to avert hospitalization. Thus, these tools serve not only as prognostic instruments but also as practical decision-making aids that guide resource allocation and personalized care plans across the continuum of care.

Telemonitoring has enabled remote collection of numerous physiologic data points from patients in their homes, providing a broader picture as compared to intermittent outpatient visits [18]. However, many early telemonitoring-based algorithms have produced high false-positive rates, limiting clinical implementation [18,19,20,21,22]. While invasive monitoring devices offer greater accuracy, there is a barrier in their widespread implementation due to cost and the increased risk of complications [23].

More recently, artificial intelligence (AI) and machine learning (ML) technologies have emerged as promising avenues for enhancing HF risk stratification. These approaches are capable of analyzing vast and complex datasets to detect patterns and generate predictive models with high precision [24,25]. By leveraging advanced algorithms and machine learning techniques, AI has the potential to enhance diagnostic accuracy, facilitate earlier detection, and support clinical decision-making. These technological innovations could significantly address the gaps in current diagnostic approaches and improve the overall management of HF, ultimately leading to better patient outcomes and more efficient healthcare delivery [26].

AI has been applied to various data modalities including EHRs, electrocardiograms (ECGs), echocardiography, and heart sounds, with several studies demonstrating diagnostic accuracies with over 90% sensitivity and specificity. In one scoping review, AI models predicting 30-day readmission in HF patients achieved AUCs ranging from 0.61 to 0.79, outperforming many conventional risk scores [27]. A recent novel innovation developed an AI model to study electrocardiogram (ECG) images and determine HF risk stratification [28]. The study found that a positive AI-ECG screen with the use of their model resulted in up to a 24-fold higher risk of new-onset HF, a finding that was further consistent when adjusted for comorbidities. This initiative can be further extrapolated to identify patients with known HF who may be at risk for hospital admissions. 

## 3. GDMT Implementation and Titration

GDMT remains the foundation of evidence-based care for patients with HF with reduced ejection fraction (HFrEF). When implemented and titrated appropriately, GDMT has been shown to reduce mortality and hospitalizations, and improve overall quality of life [29,30]. Despite well-established guidelines, large-scale registries continue to demonstrate substantial gaps in both the initiation and up-titration of recommended therapies [31].

The four primary classes of GDMT, endorsed by the 2022 American Heart Association, American College of Cardiology, and Heart Failure Society of America (AHA/ACC/HFSA) guidelines, include

1. Angiotensin receptor–neprilysin inhibitors (ARNIs): sacubitril/valsartan demonstrated a 20% relative risk reduction in cardiovascular death or HF hospitalization compared to enalapril in the PARADIGM-HF trial [32].
2. Beta-blockers (carvedilol, metoprolol succinate, and bisoprolol): these agents reduce all-cause mortality by approximately 30% [33].
3. Mineralocorticoid receptor antagonists (MRAs): spironolactone and eplerenone significantly reduce mortality and HF hospitalizations [34].
4. Sodium-glucose cotransporter 2 (SGLT2) inhibitors: dapagliflozin and empagliflozin have demonstrated reductions in HF hospitalization and cardiovascular death regardless of diabetes status [35].

Landmark clinical trials such as PARADIGM-HF [32] and DAPA-HF [35] have shown that ARNIs and SGLT2i reduce the combined risk of cardiovascular death and HF hospitalization by approximately 20–30%. Similarly, beta-blockers and MRAs have shown mortality benefits ranging from 30% to 35% [33,34]. Additional therapies such as ivabradine, hydralazine–nitrate combinations, and vericiguat may be considered in patients with persistent HF symptoms despite optimized GDMT, or those with specific comorbidities [36].

Despite this strong evidence, GDMT remains underutilized, or suboptimally dosed in real-world practice. Data from the CHAMP-HF registry indicated that fewer than 25% of eligible patients are prescribed target doses of all four pillars of GDMT, and only 40% receive all indicated therapies at any dose [31]. To address this gap, evidence-based medication optimization is a key component of HF-DMPs. To ensure success, programs aim to include mechanisms to systematically identify patients who are not receiving optimal dosages of their HF medications. These systems are then paired with pre-determined protocols for initiating and titrating therapies to evidence-based target or maximally tolerated doses [37]. Establishing standardized medication optimization guidelines and communicating them effectively across all levels of care is critical in ensuring evidence-based care for patients with HF. Patient education, an active part of HF-DMPs that is explored in further detail below, also plays a vital role in improving medication adherence and reducing all-cause rehospitalizations [38,39].

Traditionally, stepwise titration of medications has been employed; however, recent evidence supports a more aggressive approach. Rapid, simultaneous initiation and optimization of all four GDMT pillars has been associated with better outcomes compared to sequential titration. The STRONG-HF trial demonstrated that rapid initiation and aggressive up-titration within 2 weeks after hospital discharge resulted in 23 fewer patients per 1000 experiencing a composite of HF hospitalization or cardiovascular death and 7 fewer deaths compared to the conventional approach [30,40].

Protocols often recommend initiating all four classes within the first 4 weeks of diagnosis or decompensation, with titration every 2–4 weeks as tolerated, based on patient-specific factors such as blood pressure, renal function, and potassium levels [41]. The STRONG-HF trial further supported early and aggressive titration, showing a reduction in 180-day mortality and HF readmissions when therapy was initiated during hospitalization and intensified post discharge [40].

Pharmacist-led GDMT titration clinics have emerged as a successful strategy to close the implementation gap. In inpatient settings, pharmacists collaborate with multidisciplinary teams to initiate therapy during hospitalization, improving the likelihood of therapy continuation after discharge [42]. In outpatient settings, pharmacist-managed clinics enable frequent monitoring, dose adjustments, and education, which in turn facilitates safer and more efficient up-titration of GDMT [42]. Clinical trials have demonstrated that pharmacists working within multidisciplinary HFDM teams increased the proportion of patients achieving target doses by over 35%, reduced medication errors, and improved patient education [43]. Integrating pharmacists into HF care teams provides a scalable and cost-effective method for optimizing GDMT delivery and aligns with interventions aimed at HF quality improvement efforts [37,43].

## 4. Multidisciplinary Team-Based Care

Multidisciplinary team-based care is a foundational element of HF-DMPs, particularly for patients with complex diseases and frequent exacerbations. This approach spans pharmacological optimization, lifestyle support, and social needs. Studies consistently show that multidisciplinary strategies improve clinical outcomes, reduce morbidity and mortality, and enhance patient quality of life. Evidence from large meta-analyses and randomized controlled trials shows that HF-DMPs involving multidisciplinary teams reduce hospital readmission rates by 30–40% and mortality by up to 25% over 6–12 months of follow-up [30,44].

The core team members consist of diverse healthcare professionals, including HF cardiologists, nurse practitioners, clinical pharmacists, dietitians, social workers, and care coordinators, to deliver coordinated, patient-centered care. Each professional plays a critical role in the continuum of care: cardiologists and advanced practice providers oversee medical management, pharmacists guide safe and effective medication use, dietitians educate patients on dietary modifications, and social workers and coordinators support transitions of care, access to medications, and SDoH [45]. In addition to the main HFDM team, there are significant contributions by relevant subspecialists such as nephrologists, palliative care teams, and electrophysiologists. These experts provide critical input in complex cases involving renal dysfunction, refractory symptoms, arrhythmias, or advanced device management. Integrating subspecialty care ensures comprehensive decision-making, especially in patients with multimorbidity or advanced disease [46,47].

Review studies have demonstrated that programs with a higher number of intervention team members, including specialized HF cardiologists and nurses, showed significantly more success with reductions in 30-day and 90-day hospital readmissions [48]. Moreover, a systematic review of randomized controlled trials found that structured follow-up by a multidisciplinary team led to a reduction in rehospitalization risk by about 37% as compared to follow-up relying primarily on telephone contact with primary care providers [49]. Routine multidisciplinary team meetings are essential for reviewing patient progress, addressing clinical deterioration, identifying barriers to adherence, and tailoring individualized treatment strategies. These collaborative reviews promote unified decision-making and allow for timely adjustments to the care plan, improving continuity and reducing fragmentation of care [50]. They also ensure that the latest guidelines are being followed to promote consistent provision of evidence-based medical care.

Nursing-led interventions are a particularly high-yield component of the multidisciplinary framework. Nurse-led HF clinics, structured education programs, and telemonitoring initiatives have demonstrated improvements in medication adherence, patient engagement, and early recognition of decompensation. These interventions are associated with lower hospitalization rates and improved patient-reported outcomes, particularly in high-risk populations such as the elderly or those with low health literacy [38,51].

Altogether, the multidisciplinary model represents a shift from physician-centric care to a coordinated network of providers working in concert to deliver comprehensive HF management. Its integration into HF-DMPs is essential for achieving sustained improvements in outcomes and reducing healthcare utilization.

## 5. Patient Education and Self-Management Support

Patient education is at the core of heart failure disease management. HFDM is structured around patient education and self-care management. Educating patients regarding sodium restrictions, fluid restriction, medication compliance, daily weights, and monitoring of symptoms is critical for patient self-care management. In addition, patients are also educated regarding maintaining a healthy lifestyle to incorporate daily exercise and smoking cessation.

The Heart Failure Society of America (HFSA) and the American College of Cardiology (ACC) recommend restricting daily sodium intake to 2000–3000 mg, and the AHA recommends a lower limit of less than 1500 mg per day [52]. Educating patients on how to limit sodium intake in their diet requires focus on sodium restriction recommendations, foods high in sodium to be avoided (such as processed foods, prepared and frozen meals, canned soups), food labels literacy, and knowledge on terms such as low sodium, sodium-free and reduced sodium—knowledge on these topics will allow patients to make healthier sodium intake choices.

Fluid restriction education and daily weight monitoring are additional practices that patients need to understand for self-care management. It is important to note that the ACC/AHA/HFSA guidelines for HF generally recommend fluid restriction for patients with severe HF to help manage symptoms and fluid overload. These guidelines acknowledge that fluid restriction is commonly recommended, but the evidence in this area is of low quality [53]. The most recommended fluid restriction for patients with stage “C” and “D” HF is 1.5–2 liters per day. This restriction can be individualized for specific patients depending on their clinical status. The FRESH-UP study noted that patients with stable HF who did not have a HF hospitalization for 6 months or more could be more liberal with their fluid intake. However, patients with recent admissions for acute decompensated HF and those in the post-discharge phase were required to be stricter with their fluid restrictions [54].

Patients are also taught to include daily weight measurement in their routine. A weight gain of 2–3 pounds overnight or 5 pounds over one week is an indication of potential fluid accumulation. Patients are taught to weigh themselves daily after their first morning void, before breakfast, while wearing the same amount of clothing, and on the same scale. This specific way of daily weighing allows them to identify their “dry” weight and the weight gain that is used when teaching patients to report weight gain overnight or in one week. Their dry weight can change as their flesh weight increases or decreases. Utilizing visual aids helps patients with these recommendations, providing tools for patients to understand these practices to help identify and prevent the onset of an exacerbation of decompensation.

**Figure 2:**
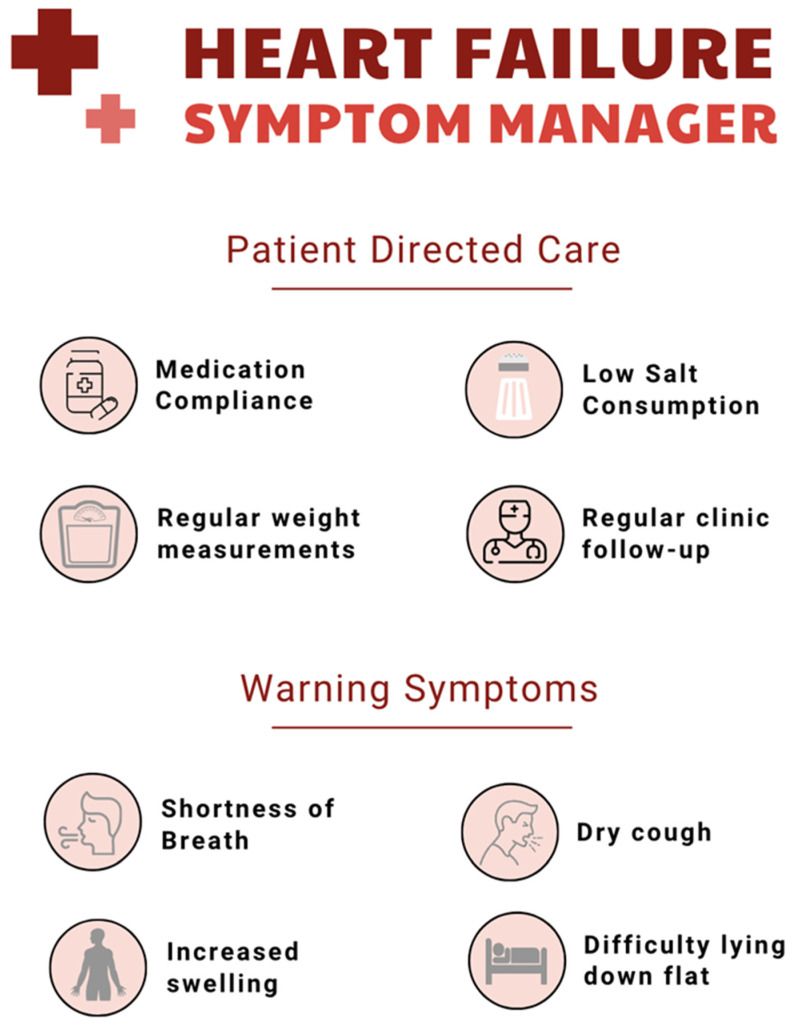
An overview of patient-directed management of heart failure and associated warning symptoms.

Symptom recognition is emphasized as the master key to identifying the onset of an exacerbation. Patients are taught the signs and symptoms, which include changes in shortness of breath, weight changes, changes in energy level, and sleeping issues. Patients are instructed how to implement patient self-care diuretic adjustments and when to call for worsening symptoms. The patients are taught using a visual aid self-check plan that is color-coded with symptoms, noting specific symptoms to pay attention to and symptoms that require medical attention for evaluation [55]. Figure 2 provides an overview of a symptoms tracker that can help HF patients with self-management.

Another integral aspect of patient self-care management is medication adherence. Patients are counseled regarding the importance of taking medications as directed. This education includes medication regimen examples with specific time frames and incorporation of tools that can be utilized to help with adherence, such as using a pill box and setting reminders on cell phones. Patient counseling includes informing them of potential medication side effects, such as fatigue or dizziness, and the importance of reporting these symptoms.

Given the thorough nature of these self-care management needs, it is essential to ensure that patients understand all these steps. A key technique used is the “teach back” method of education, which involves asking the patient to explain what they have learned from the healthcare provider in their own words. This method allows for immediate re-education or clarification of any misunderstandings and ensures that the patient properly understands the educational material that was taught. This process should be continued until the patient correctly recalls the information and is known to improve knowledge, skills, and self-care abilities in patients with chronic disease [56,57]. Surveys are another method that is used to assess a patient’s knowledge regarding their HF management; pre- and post-education surveys are a tool that can measure a patient’s understanding of how to manage their chronic disease. The survey should also be in plain language with short sentences to assess the patient’s knowledge before and after the education session.

Health literacy must also be taken into consideration when educating patients utilizing printed teaching material and tools. The reading material and tools should use plain language that is easy to understand, short sentences, and visual aids. Health literacy-adjusted materials will assist the patient’s understanding to be able to use the information they have learned to manage their disease process [58].

In addition to this, patient education modules are structured programs with education modules that are easy for patients to understand and can be accessed for free. Structured education modules are a valuable resource that can be utilized to provide education for patients, their caregivers, and their families. There are also animation videos and colorful pictures that are easy to understand for those with lower literacy rates [55].

Patient lifestyle education needs to include physical activity material, which includes increasing activity to 150 min a week of moderate-intensity aerobic activity, 75 min per week of vigorous aerobic activity, or a combination of both, preferably spread throughout the week. It is important to inform patients that this can be performed in small spurts throughout the day. Patent education should also include moderate- to high-intensity muscle-strengthening activity (such as resistance or weights) at least 2 days per week. Lifestyle modules for exercise can also be found on the AHA website [55].

Patient behavioral education includes materials pertaining to smoking cessation. Patients need to be supported and encouraged to start their journey to quit smoking. Many pulmonary departments have smoking cessation programs that are free and provide smoking cessation tips as well as information on smoking cessation medications. The Quit Now phone number (1-800-Quit-Now) is another free tool for smoking cessation, which provides telephone-based tobacco cessation counseling services in various different languages throughout the USA [59].

## 6. Transitional Care and Post-Hospitalization Follow-Up

Patients admitted for an initial diagnosis of HF or an acute exacerbation of known HF require a smooth transition to outpatient care and close follow-up. A HF-DMP provides this transition, and its first step to establishing post-discharge care is to ensure that all patients are given a follow-up appointment with the date and time provided prior to their discharge and clearly outlined in their discharge instructions. The program also confirms that patients have all their medications on discharge by having them delivered directly to their inpatient rooms through an initiative known as “Meds to Bed”. A HF-DMP provides early communication by ensuring a phone call is made to the patient within 3 days of discharge by the HF nurse navigator—the purpose of this call is to confirm that patients are taking their medications as prescribed and are reminded of their follow-up outpatient appointment. This phone call also addresses transportation issues to ensure that the patient is able to come to their appointments. The HF nurse practitioner follows the patient closely by making assessments, titrating GDMT and diuretics if necessary, and continuing the educational process for patients in the HF-DMP at every visit. Specific vulnerable patient populations will require frequent follow-up visits by the nurse practitioner or HF physician, which can be as frequent as weekly appointments, to prevent HF exacerbations, which can require emergency department visits and hospital admissions.

Utilizing outpatient infusion centers provides another resource for patients in acute exacerbations of HF. It has been shown that outpatient IV diuresis is well tolerated, effective, and should be considered as a strategy [60]. Patients who require additional diuretic therapy beyond their oral dose can be referred to the center once or twice a week. This process also allows for monitoring of electrolytes to ensure patient safety to prevent potential arrhythmias from electrolyte imbalances. If necessary, patients can also receive potassium at the IV infusion center.

## 7. Remote Monitoring and Telehealth

Telehealth and telemonitoring have emerged as valuable components of medical care and play an important role in the development of HF-DMPs. In an overview of telehealth in CVD management, the AHA highlighted that telehealth could be useful for medication adherence, risk factor modification, and symptom monitoring in both coronary artery disease and congestive HF [61]. This may be accomplished through direct patient surveys and recording of blood pressure, smoking frequency, dietary salt intake, and weight measurements. Data collection alongside telehealth check-ins can result in continuous fine tuning of medication regimens and lifestyle alterations. Additionally, this modality can serve in reducing transportation costs for rural and low-income populations while improving access to care for elderly patients who may face difficulty attending an increasing number of medical appointments [62].

Recent studies have examined both invasive and non-invasive telemonitoring methods for effectiveness in management of HF patients. A meta-analysis of RCTs and observational studies published in 2023 looked at the current data in regard to both these modalities and their effects on mortality and HF hospitalization. Overall, they found a significant 16% reduction in all-cause mortality and a significant 19% reduction in first HF hospitalization. Subgroup analysis also showed that non-invasive methods of telemonitoring showed significant reductions in all-cause mortality, first HF hospitalization, and total HF hospitalizations. Non-invasive modalities included telemonitoring with biometric data collection, structured telephone support, and complex telemonitoring, which combined components of the latter two [63].

Additionally, the TIM-HF2 trial performed from 2013 to 2017 highlighted a significantly reduced percentage of days lost due to unplanned cardiovascular hospitalizations and significantly reduced all-cause mortality in patients with NYHA class II or III HF who were enrolled in a structured remote patient management program. Core components of this program included daily measurement of weight, blood pressure, single-lead ECG, heart rate, and oxygen saturation. This was combined with a telemedicine staff of nurses and physicians available 24/7 to review the transmitted data [64].

More invasive monitoring techniques have also shown promise, driven by the understanding that hemodynamic congestion often precedes clinical symptoms. Most notable is the CardioMEMS device by Abbott, (Plymouth, MN 55442) which showed a 30% reduction in HF hospitalizations in patients with moderate-to-severe HF in the CHAMPION trial through invasive monitoring of PA pressure. This led to its’ FDA approval and European Conformity mark to reduce HF hospitalizations [65].

Beyond pulmonary artery sensing, research has also been extended to other modes of monitoring, including RV pressure and LA pressure. In the COMPASS-HF trial published in 2008, an implantable RV pressure sensor named Chronicle IHM used in patients with NYHA class III and IV HF showed a nonsignificant reduction of 21% in HF-related events. This was performed after a smaller study had shown promise with a significant 57% reduction in HF hospitalizations [66]. Invasive monitoring of LA pressure has also been investigated in patients with chronic HF through the LAPTOP-HF trial. The study, however, was stopped prematurely due to procedure-related complications and safety concerns. Although a final endpoint could not be reached, an analysis of 486 of the total 730 patients showed a significant 41% reduction in HF hospitalizations at 12 months, highlighting the potential benefit direct LAP sensing could offer with future safety optimization [65].

While the evidence for a positive role non-invasive and invasive telemonitoring can play in HF disease management continues to develop, recent clinical guidance has also emphasized tailoring strategies based on a patient’s individual disease severity and stability. Patients with early-stage HF or improving HF may require more long-term surveillance to alert to deviations from stability, whereas patients with more recent HF decompensation may need more intensive monitoring [67]. Consequently, when establishing a telehealth component to a HF disease management program, stratifying the approach based on disease severity should be considered. 

Ensuring patient engagement with the data collected is also an important point to consider in incorporating telemonitoring. In a 2023 statement, the AHA emphasized that health data collected should be accessible to patients. This will allow for transparency, promote insight into health progress, and potentially lead to strengthened compliance to current medication regimens [68]. Mobile-based applications offer a promising solution with the ability for patients to see their health trends over time. 

## 8. Performance Metrics and QI

Despite advances in guideline-directed medical therapy (GDMT), care delivery and adherence to these guidelines vary among institutions. To bridge this gap, national organizations have developed performance metrics to standardize, assess, and improve the quality of HF care. These metrics serve as benchmarks for pay-for-performance initiatives, public reporting, and internal quality improvement.

There are various types of performance metrics in HF, with the majority focusing on three categories: process, outcomes, and structural measures.

Process measures evaluate the actions performed by clinicians. These actions are dictated by guidelines and current practice recommendations by various societies like the AHA, ACC, and Centers for Medicare & Medicaid Services. In patients with signs and symptoms of HF, the ACC/AHA/HFSA 2022 guidelines recommend an assessment of left ventricular ejection fraction to determine the HF phenotype (HFrEF, HFmrEF, or HFpEF) and to guide therapy choices, including eligibility for GDMT, device therapies, and prognosis estimation [29,69].

Outcome measures are critical indicators used to evaluate the effectiveness of H management by assessing the results of care as experienced by the patient. Unlike process measures that evaluate actions taken, outcome measures reflect the actual health status, survival, quality of life, and healthcare utilization following treatment. These measures help stakeholders—including clinicians, health systems, payers, and regulatory bodies—understand whether interventions lead to meaningful improvements in patient outcomes. Thirty-day readmission rate, mortality, and self-reported outcomes utilizing the Kansas City Cardiomyopathy Questionnaire have been utilized in these metrics [55,70,71].

Structural measures in HF evaluate the infrastructure, staffing, organization, and systems that support the consistent delivery of evidence-based care. Structural metrics assess the foundational capabilities required to provide high-quality care.

These measures are particularly important in HF management, where multidisciplinary teams, access to advanced therapies, and care coordination infrastructure significantly influence patient outcomes. This is clearly documented in the HFSA comprehensive HFDM position statement [72] and in the scientific statement from the AHA regarding outpatient care of HF patients [9].

Table 1 below summarizes the key HF performance metrics commonly used by professional societies and federal agencies.

There are multiple challenges in implementing these metrics. Performance metrics in HF have evolved from process-oriented checklists to more nuanced measures that reflect patient outcomes, care structure, and equity. Data collection has been a burden, as manual chart abstraction remains a barrier in many settings requiring more resources. In addition, metrics like readmission rates can unfairly penalize safety-net hospitals. 

Ongoing refinement and integration of digital tools, PROMs, and socioeconomic contexts are essential to ensure metrics truly reflect quality and improve outcomes in HF. 

## 9. Social Determinants of Health

HF related hospital re-admissions within 1 year can be as high as 50% and are heavily impacted by SDoH such as access to healthcare, poverty, health literacy, substance use, food insecurity, and poor housing [77,78]. In order to address these challenges, HF-DMPs are utilized to directly support unique patient needs. As proposed by the AHA, a DMP comprises six different elements, including the following: population identification processes, evidenced-based guidelines, collaborative practice models, patient self-management guidelines, process and outcome management, and reporting and feedback loops [78]. DMPs screen patients for disparities in access to care via interdisciplinary collaboration. Some screening tools include the Accountable Health Communities (AHC) Health-Related Social Needs (HRSNs) Screening Tool and Protocol for Responding to and Assessing Patient Assets, Risks, and Experiences (PREPARE). These screening tools cover questions regarding living situation, food, transportation, utilities, safety, financial strain, employment, family/community support, education, physical activity, substance use, mental health, and disabilities.

Complex management of HF includes the incorporation of quadruple GDMT. Patients often face barriers in acquiring these medications such as out-of-pocket medication costs, insurance access, transportation to appointments, and adherence to polypharmacy, all of which contribute to lower rates of optimal GDMT therapy [79]. Interventions such as social work assistance for Medicaid and Medicare applications and renewals are necessary to mitigate healthcare coverage-related barriers to treatment. Case management also assists with evaluations of patient eligibility for prescription drug coverage via patient assistance programs [80].

Partnership with community health workers is particularly effective in helping patients navigate issues like poverty and homelessness [80] by providing referrals to housing assistance programs. These patients often live around food deserts or have limited activity due to low neighborhood safety. Targeted interventions with the support of dietitians can help locate local community food pantries or tailor the prescribed low-sodium diet to cultural preferences [80].

Overall, the use of patient care navigators and community health workers (CHWs) can act as peer-driven assistance for patients without adequate social support from family or friends. They help guide patient self-management and encourage autonomy by assisting with medication reconciliation, putting together pill boxes at discharge, and organizing follow-up appointments [80]. Making home visits and arranging medical transportation for appointments are other crucial roles played by CHWs for patients who otherwise cannot financially afford it [80]. Additionally, remote monitoring devices such as weight scales and blood pressure monitors are also useful for patients in underserved areas. Poor health literacy is another notable risk factor in healthcare disparities that is addressed by DMPs. Nurses assist with explaining educational material that is provided in the patient’s primary language and in plain non-medical terms as well as utilizing the teach back method to ensure their comprehension. The AHA also encourages the use of visual aids to assist with memory retention and language that reflects a fifth-grade or lower reading level [80]. Furthermore, DMPs screen for mental health conditions such as depression and anxiety common in patients navigating chronic health failure by referring to behavioral health and therapy resources. The programs improve patient quality of life and reduce hospital readmissions by mitigating barriers to care.

## 10. Challenges and Limitations

While HF-DMPs are increasingly recognized as a foundation of high-quality chronic care, their scalability and sustainability remain limited by resource constraints and implementation challenges. These programs depend on substantial institutional infra-structure, reliable funding, and a trained multidisciplinary workforce—elements that are not universally available.

Rural and underserved populations are particularly affected by these disparities. Ac-cording to the Get with the Guidelines Heart Failure registry, patients hospitalized for HF at rural hospitals were independently associated with lower use of some guide-line-recommended therapies at discharge [81]. In addition, the digital divide poses a barrier to implementing remote monitoring and telehealth strategies; a recent study noted that only 61% of adults over age 65 (a population heavily represented in HF care) report comfort with digital technologies, and disparities are more pronounced among racial and ethnic minorities [82].

Although artificial intelligence (AI) and implantable monitoring devices like CardioMEMS have been shown to reduce HF hospitalizations and improve quality of life [4], these tools require integrated electronic health systems, payer support, and patient engagement—factors that vary widely across health systems. Without addressing these systemic gaps, the adoption of these innovations may risk exacerbating existing healthcare inequities [83].

## 11. Conclusions

Heart Failure Disease Management Programs (HF-DMPs) are essential in addressing the rising complexity and healthcare burden of heart failure. Through standardized processes encompassing risk stratification, aggressive GDMT titration, multidisciplinary team care, structured education, and integration of telehealth and social services, these programs can significantly improve patient-centered outcomes. While implementation varies across healthcare systems, the adoption of structured and scalable HF-DMPs can reduce readmissions, enhance quality of life, and ensure delivery of evidence-based care across diverse populations. Strengthening these programs with digital health tools and equity-focused strategies will be critical in closing gaps in care.

## 12. Future Directions

As the field of heart failure management evolves, several future directions are anticipated for HF-DMPs:

- Integration of artificial intelligence (AI): AI-powered risk prediction models and ECG interpretation tools may enable real-time, personalized interventions.
- Expansion of virtual care: enhanced telemonitoring and remote titration clinics will increase access, especially for rural and underserved populations.
- Equity-driven customization: tailoring HF-DMPs to address disparities in access, health literacy, and social needs will be essential to achieving health equity.
- Outcomes-based research: large-scale studies are needed to evaluate the long-term impact of HF-DMPs on mortality, quality of life, and cost-effectiveness across various healthcare settings.
- National benchmarking: widespread adoption of performance metrics will allow for standardized comparisons and continuous quality improvement efforts.
- Rural health focus: investment in rural telehealth infrastructure, culturally sensitive patient education, and policy incentives will augment sustainable multidisciplinary care delivery.

## Figures and Tables

**Table 1:** A summary of key heart failure performance metrics commonly used by professional societies and federal agencies.

Category	Metric	Description	Organization	References
Process	Documentation of LVEF	Percent of HF patients with documented assessment of LVEF	AHA/ACC, CMS	[29,69]
Process	GDMT Prescription	ACEi/ARB/ARNI and beta-blocker prescribed in HFrEF	AHA/ACC, CMS	[29,55,69]
Process	Aldosterone Antagonist Use	For patients with LVEF ≤ 35%, if no contraindications	ACC/AHA/HFSA	[29,55,69]
Outcome	30-Day Readmission Rate	Risk-adjusted rate of unplanned readmission after HF discharge	CMS Hospital Compare	[55,73]
Outcome	In-hospital Mortality	All-cause inpatient mortality in HF hospitalizations	CMS, Vizient	[69,70]
Outcome	Health Status/Quality of Life	Patient-reported outcomes using tools like KCCQ	PCORI	[71,72]
Structural	Presence of HF Program	Availability of multidisciplinary HF disease management team	HFSA	[74]
Structural	Access to Advanced HF Therapies	Transplant, LVAD, and palliative services	UNOS, HFSA	[29,75,76]

Abbreviations: AHA—American Heart Association; ACC—American College of Cardiology; CMS—Centers for Medicare & Medicaid Services; HFSA—Heart Failure Society of America; KCCQ—Kansas City Cardiomyopathy Questionnaire; PCORI—Patient-Centered Outcomes Research Institute; UNOS—United Network for Organ Sharing.
